# Supplementary material for: Learning curve and surgical outcome of robotic assisted colorectal surgery with ERAS program
Source: Sci Rep. 2022 Nov 29;12:20566. doi: 10.1038/s41598-022-24665-w (PMC9709162; doi:10.1038/s41598-022-24665-w)
Supplement: Supplementary file 2 — Supplementary Information 2. [file 41598_2022_24665_MOESM2_ESM.zip › RAL Raw data-Ñ[▒K/105/HS_ñw╝╨░O▒Kñσ.pdf]

正式摘要(電子簽章：000000223603736)		
W92 / W92 ☐ 英文標題(注意:無英文姓名-請補病人基本檔!)	☐【讀取】憑證	

臺中榮民總醫院

出院病歷摘要

轉入號 : 轉出號 :

[redacted]

(8)流水編號	
(9)入院日期	110/08/17 (2021) 直腸外科 W92 - 37
(10)轉科日期	
(11)出院日期	110/08/25 (2021) 住院天數計 8 日

診斷

- 入院 :1. Acute appendicitis, s/p laparoscopic appendectomy on 2021/07/13, pathology: appendix mucinous adenocarcinoma, pT4aN0M0.
- 出院 :1. Acute appendicitis, s/p laparoscopic appendectomy on 2021/07/13, pathology: appendix mucinous adenocarcinoma, pT4aN0M0, s/p robotic right hemicolectomy on 2021/08/18

主訴

- Lower abdominal pain for 1 month

病史

- This 60 y/o female patient had denied systematic disease history.

  She had right lower abdominal pain, and visited PuLi branch of TCVGH. Acute appendicitis was noted, and laparoscopic appendectomy was done on 2021/07/13. However, pathology revealed appendix mucinous adenocarcinoma, pT4aN0M0.
  Under the impression of appenix malignancy, she was admitted for surgery.

體檢發現

- CEA 1.6

  2021 CT: apen ca.
  2021/8/5 lung CT: no mets.
  - General appearance :
    - easy looking , conscious clear , without cardiopulmonary distress
    - Vital signs自動帶入date 體溫(BT) 脈搏(P) 呼吸(R) 血壓(BP) 身高(BH) 體重(BW) BMI
      20210817 37.1C 100/min 18/min 157/95mmHg 150.7cm 61.8kg 27.2
    - Vital signs: B.P. 157/95 mmHg , P.R. 100 /min , R.R. 18 /min , B.T. 37.1 ℃
  - HEENT :
    - Eyes: no anemic conjunctiva, no icteric sclera, normal EOM
    - ENT: no otorrhea, no rhinorrhea, no erythematous mucosa, no injected tonsil, no deformity
  - Neck: supple, freely movable, trachea midline,no deformity, no engorged jugular vein , no palpable thyroid or lymph node enlargement
  - Chest:
    - Inspection: Grossly normal, symmetric and fully expansion, no accessory muscle used, no operation scar, no spider angiomata, no gynecomastia
    - Palpation: no palpable mass or nodule, no subcutaneous emphysema, no tenderness
    - Ausculation: clear breathing sound
  - Heart:
    - Palpation: no thrill, no heave
    - Percussion: normal heart size
    - Ausculation: regular heart beat, normal S1, S2, no gallop, no murmur
  - Extremities: No deformity, full ranged of motion without limitation, no clubbing finger or nail cyanosis, no pitting edema, no ulcer or pigmentation, no obvious skin lesion, no

- varicose vein, no bone, joint or muscle tenderness

CRS CONDITION

- Karnofsky performance status: (100) Normal, no complaints, no evidence of disease
- PE of Abdomen:
- CEA: 1.60 ng/ml
- Imaging Studies:
- Colonoscope report: nil
- Sono , CT of abdomen and chest Films:nil
- Figures:
  1. colon & rectum

手術日期及方法

- 手術日期及方法20210818 Robot Assisted Colectomy(R)

住院治療經過

- After surgery, she had abdomen distension on POD2, and arranged NPO plus KUB follow up. Evac was given for stool passage. She now can take diet regularily. She is discharged today.

合併症

一般檢查

- 累積報告 :

DATE	TIME	ALB	TP	BIL.T	BIL.D	ALKP	AST	ALT	LDH	AMY	LIPASE	NH3	Ferritin	UA
1100818	1338	3.7		0.40	0.09	107	23	22						

DATE	TIME	WBC	RBC	HGB	HCT	MCV	PLT	BLST	NEUT	BAND	SEG	LYM	MON	EOS	BAS
1100818	1339	11760	3.82	11.8	34.3	89.8	269		92.4			6.2	1.0	0.1	0.3

DATE	TIME	NA	K	CL	BUN	CREAT	eGFR	CA	Mg	P	CRP	ESR	PCT	D-Dimer	LACTATE
1100818	1338	142	4.3	109	9	0.77	81.27	8.2							

DATE	TIME	GLUCOSE	HbA1C	CHOL	TG	HDL	LDL	KETONE	CK	CKMB	TRO-T	PT-P	APTT-P	NT-pro BNP
1100818	1338	157												

特殊檢查

病理報告

- pending

其它

出院時情況

- 15.計劃性出院

出院指示

藥名	劑量	劑量單位	用法	頻次	數量	數量單位	現況	天數	單位
Voren-K tab 25mg	1	TAB	PO	TID	15	TAB	TH	5	天
Meclizine tab 25mg	1	TAB	PO	TID	15	TAB	TH	5	天
(口)Takepron OD tab 30mg	1	TAB	PO	QDAC	5	TAB	TH	5	天
Mag. Oxide tab	2	TAB	PO	TID	30	TAB	TH	5	天
Ultracet tab	1	TAB	PO	QID	20	TAB	TH	5	天
Cividoid gel 20g		TUB	TOP	QD	0	TUB	TH	1	瓶
100-Neurontin cap	1	CAP	PO	TID	15	CAP	TH	5	天
Xanax tab 0.5mg	1	TAB	PO	QNPRN	5	TAB	TH	5	天

出院計劃

主治醫師 2222H 林俊余 / 住院醫師 2340H 郭連雄

正式摘要(**電子簽章：000000223603736)**

W92 / W92

doctorRank:B
